# Left Shifting of Language Related Activity Induced by Bihemispheric tDCS in Postacute Aphasia Following Stroke

**DOI:** 10.3389/fnins.2019.00295

**Published:** 2019-04-26

**Authors:** Sarah Feil, Peter Eisenhut, Frauke Strakeljahn, Sarah Müller, Claude Nauer, Jens Bansi, Stefan Weber, Alexandra Liebs, Jean-Pascal Lefaucheur, Jürg Kesselring, Roman Gonzenbach, Veit Mylius

**Affiliations:** ^1^Department of Neurology, Center for Neurorehabilitation, Valens, Switzerland; ^2^Schweizer Hochschule für Logopädie Rorschach, Rorschach, Switzerland; ^3^Department of Radiology, Kantonsspital Graubünden, Chur, Switzerland; ^4^Service de Physiologie – Explorations Fonctionnelles, Hôpital Henri-Mondor AP-HP, Université Paris-Est Créteil, Créteil, France; ^5^Department of Neurology, Kantonsspital St. Gallen, St. Gallen, Switzerland; ^6^Department of Neurology, Philipps-University Marburg, Marburg, Germany

**Keywords:** transcranial direct current stimulation, aphasia, brain stimulation, language therapy, picture naming task

## Abstract

Both anodal transcranial direct current stimulation (tDCS) of the left IFG and cathodal stimulation of the right IFG were shown to improve rehabilitation of stroke patients with Broca’s aphasia. The study aimed at assessing the impact of a bihemispheric IFG stimulation compared to sham on postacute non-fluent aphasia. Twelve patients with non-fluent aphasia were included at least 4 weeks following cerebral stroke. Ten daily sessions of 2 mA bihemispheric verum or sham tDCS (anode on left IFG and cathode on right IFG) were performed concomitantly with individual language therapy in a double-blinded randomized controlled study with parallel group design. Language functions [i.e., communication (ANELT), picture naming and the Aachen aphasia test (AAT)] were assessed up to 1 month following tDCS. The picture naming task significantly improved (increased number of nouns) at the end of the tDCS procedure in the verum but not sham group. Improvements in the picture naming task and the communication task of the AAT at 4 weeks after tDCS procedure were only seen in the verum group. In patients with postacute cerebral stroke, repeated sessions of tDCS applied on both IFG concomitantly with language therapy were able to induce immediate effects on picture naming presumably due to an early left shift of language-associated function that maintained for 4 weeks. Effects on clinically relevant communicative abilities are likely.

## Introduction

Aphasia is a relevant sequela for a large proportion of patients after cerebral stroke (Engelter et al., 2006). Language therapy in aphasia recently focused on the rehabilitation of communicative abilities with conversational therapy. However, further improvements are needed to ameliorate the clinical benefit provided by speech and language therapy (Basso, 2010). In the recent years, the add-on use of non-invasive cortical stimulation gained interest in this context, as highlighted by international recommendations (Lefaucheur et al., 2014, 2017). However, neither repetitive transcranial magnetic stimulation (rTMS) nor transcranial direct current stimulation (tDCS) are still recommended in daily practice, since the level of evidence is lowered by the heterogeneity of study quality, methods of stimulation or evaluation, clinical profiles of aphasic syndromes, or the delay of stimulation time after stroke onset.

Cortical activity is thought to be dramatically reduced after stroke within the affected region [e.g., left inferior frontal gyrus (IFG) in non-fluent aphasia] and then to increase in the contralateral homonym cortical area (e.g., right IFG) because of a decreased transcallosal inhibition from the affected to the unaffected hemisphere (Hamilton et al., 2011). This is in line with the theory of post-stroke interhemispheric rivalry (Kinsbourne, 1977). However, it remains debated whether the increased contralesional activity leads to enhance the defective activity in the stroke lesion area before adjacent cortical regions progressively regain function in the affected hemisphere in the four to 12 months following stroke onset (Mylius et al., 2012). Thus, the time point of cortical stimulation is assumed to be critical with respect to natural stroke recovery. Although rTMS studies in chronic and post-acute aphasia (Barwood et al., 2011; Weiduschat et al., 2011) suggest that a decrease of cortical excitability on the right IFG reverses dysfunctional cortical activity, the exact temporal course of cortical reorganization is not known so far and is supposed to vary depending on stroke severity (Saur et al., 2006).

In this work, we tried to limit the impact of natural stroke recovery processes by performing cortical stimulation at least 1 month after the occurrence of stroke. We delivered tDCS over the both hemispheres, aiming at concomitantly increasing left IFG excitability with anodal stimulation and decreasing right IFG excitability with cathodal stimulation. Previous studies of bihemispheric tDCS effects in chronic aphasia suggested that the benefits were more pronounced on multiple language-related domains (Marangolo et al., 2014, 2016). Another critical point is the concomitant application of tDCS with language therapy aiming at enhancing neuronal plasticity as suggested in recent tDCS studies (Marangolo et al., 2013a,b, 2016) following Donald Hebb’s theory on synaptic plasticity (Hebb, 1949). We further assume that the concurrent language therapy already activates neuronal subpopulation within the language network leading to increase the susceptibility of the targeted neurons for cortical stimulation (Romei et al., 2016). For example, it was shown that the resting neuronal activity of the targeted cortical area was critical for excitatory tDCS to promote motor rehabilitation (Bortoletto et al., 2015).

In summary, the present study aimed at improving non-fluent aphasia in the postacute stroke period by bihemispheric tDCS of the IFG applied concomitantly with speech therapy. We assumed that tDCS therapy, at an early stage after stroke onset, may promote left-shifting restoration of cortical activities with beneficial effects on communicative abilities in the long term.

## Materials and Methods

### Patients

Twelve right-handed patients with German or Swiss German as primary language were included at least 4 weeks up to 3 months after a first symptomatic left hemispheric stroke leading to non-fluent aphasia of intermediate severity (Supplementary Table A.1 and Supplementary Figure A.1). Aphasia was defined on behalf of the Aachen aphasia test (AAT). Only patients with anomia as a main syndrome and with slightly affected comprehension were included (comprehension of the study protocol was required).

We did not classify the patients in aphasia-syndromes, because we preferred to generate individual linguistic profiles of the patients to plan individual therapy. Patients classified into aphasic syndromes by the Aachen aphasia test, especially in this early phase, often show a heterogenous language disabilities and syndrome classification often does not help to plan individual therapy (Tesak, 2006). The study was conducted from 2015 to 2017 at the Center for Neurorehabilitation in Valens, Switzerland. Handedness was assessed with the Edinburgh handedness inventory (Oldfield, 1971). Patients with intracranial metal particles or implants, previous history of seizures, former brain surgery, apraxia of speech, dementia, cooperation difficulties, neglect, hemianopia, or further neuropsychological deficit were excluded from participation. This study was carried out in accordance with the recommendations of Good Clinical Practice (ICH-GCP) with written informed consent from all subjects. All subjects gave written informed consent in accordance with the Declaration of Helsinki. The protocol was approved by Ethikkommission Ostschweiz.

### Experimental Protocol

Sample size was estimated according to a previous bihemispheric tDCS study in post-stroke aphasia (Marangolo et al., 2013a). After inclusion, patients were randomized into blocks receiving either verum or sham tDCS (BiAS 10 for Windows, epsilon-Verlag GbR, Hochheim, Germany). Cortical stimulation was performed for 10 days (5 days during the first 2 weeks) and clinical assessment was performed 3 days before the first tDCS session (T0), 3 days after the last tDCS session (T1), and 4 weeks after the stimulation period (week 6, T2) (Supplementary Figure A.2). The primary endpoint was the performance at the picture naming task of the Aachener Aphasia Test (AAT) at T2 compared to T0 in the verum tDCS group.

Therapists and patients were blinded to the type of stimulation on behalf of a protection board between the tDCS stimulator and the therapy setting. For language test scoring, evaluation was done by a therapist that has not performed the respective testing. Stimulation was executed by a member of our lab who was not involved in the evaluation or treatment of the patient (VM or PE). Blinding of therapists, and patients was maintained until the end of the study.

### Transcranial Direct Current Stimulation

According to block-wise randomization, six patients were allocated to verum tDCS and six to sham tDCS. TDCS was delivered by a battery-driven, constant current stimulator (neuroConn GmbH, Ilmenau, Germany) through a pair of saline-soaked sponge square electrodes with a surface of 35 cm^2^. The IFG was stimulated with the anode placed over F5 and the cathode placed over F6 position of the international 10–20 EEG system for electrode placement, according to classical neuroimaging references and previous studies (Naeser et al., 2010). Stimulation intensity was set at 2 mA for 20 min for 10 sessions (Andre et al., 2016; Marangolo et al., 2016), a protocol that showed greater effects than 1 mA for 10 min for 15 sessions (Polanowska et al., 2013a,b). The sham procedure was performed as usual, by turning off the stimulator after the patients felt the initial tingling sensation for 8 s (Nitsche et al., 2003).

### Speech Therapy

All patients received 30–45 min personalized speech and language therapy two times a day (5 days a week) during the 2 weeks of tDCS application by a certified speech and language therapist blinded to randomization. However, after leaving the Center for Neurorehabilitation in Valens, the frequency of speech and language therapy sessions was not controlled and varied between individuals. Overall, patients in urban areas received more sessions than those living in rural areas.

### Picture Naming Task

Since usual German naming tasks [e.g., the German version of the Boston Naming Test (Kaplan et al., 1983) or the Wortproduktionsprüfung (Blanken et al., 1999)] only examine the ability of naming nouns, we developed our own naming task including 60 pictures of objects (nouns) and activities of daily life (verbs) taken from Pictures Cards for Language Therapy (Objects) (2000) and Pictures Cards for Language Therapy (Verbs) (2012). The Frequency classes of nouns and verbs (12.2 ± 2.26 and 12.3 ± 3.36, respectively) were controlled and determined from the Leipzig corpora collection (Database for Words, 2011). The pictures were presented grouped (first nouns then verbs) in a different order on a computer screen. Patients were requested to name the presented object or activity; no hints were given. For rating, the last pronounced words to each picture were taken into account. The pronounced words were considered right if recognizable according the AAT criteria (2/3 of the word has to be phonological correct) (Huber et al., 1983). The verbs were considered right irrespective of the correctness of the morphological form or the correctness of the added noun. The patients passed the naming task at T0, T1, and T2.

### Aachen Aphasia Test (AAT)

The AAT evaluates language function in different modalities: repeating, naming, understanding, writing, and analyzing 30 phrases of spontaneous speech (Huber et al., 1983). The token test which allows to discriminate patients with and without aphasia represents the first part of the AAT (De Renzi and Faglioni, 1978). The data was computed with the AATP program (including the calculation of *t*-values for statistical analyses) (Willmes and Guillot, 1995–2008). All subtests of the AAT were assessed at T0 and T2 by a certified speech and language therapist blinded to the randomization.

### Amsterdam Nijmegen Everyday Language Test (ANELT)

The ANELT evaluates the ability to communicate independently from grammatical and semantic correctness in daily situations (e.g., at the hairdresser) with a score from 0 to 4 (Blomert and Buslach, 1994). A German version of the ANELT with two sets of randomly presented items (1–2 or 2–1) was assessed at T0 and T2 by a certified speech and language therapist blinded to randomization. The verbal expressions were rated by another certified speech and language therapist (only the auditory component was rated).

### Assessment of Alertness

The alertness task generated by the TAP software version 2.0 was used to assess attentional control (Zimmermann and Fimm, 1995), which can be modulated by tDCS (Kang et al., 2009; Andre et al., 2016). During this task the patients had to press a button as soon as a white cross appears in random intervals on a black computer screen. Reaction times to this task were recorded at T0 and T2.

### Analysis of Lesion Size

An independent neuroradiologist blinded to the treatment groups evaluated lesion sizes according to the neuroimaging data available from the acute stage. The respective lesion size was estimated in the axial, coronal, and sagittal slices of the MRI or scanner data (in four patients only scanner data was available) in three dimensions as shown in Supplementary Figures A.4, A.5. Due to differences of the imaging sources and the lack of 3DMPRAGE sequences in half of the patients no volumetric analyses could be performed.

### Statistical Analysis

The Statistic Package for Social Science (SPSS) version 20 was used for statistical analyses. Since all parameters had a normal distribution (Kolmogorov Smirnov testing) parametric testing was computed. Between-group and within-group differences were assessed using univariate and multivariate ANOVA with repeated measures, respectively. Greenhouse-Geisser correction was used when sphericity cannot be assumed. Baseline differences as well as between- and within-group differences were assessed by using the t-test for dependent or independent variables. A p-value less than 0.05 were considered significant. The results were expressed as the mean ± standard deviation.

## Results

In the 12 patients who completed the study, no severe adverse events occur. One patient in the sham group attributed his sleeping disorder at the first evening to the first stimulation. The patients were asked to tell whether the stimulation they received was verum or not and there was no difference in patients’ reply between the verum and the sham groups.

The verum tDCS group consists of four men and two women and the sham tDCS group of six men. The patients aged between 44 and 82 years. At baseline, there was no significant difference between the two tDCS groups regarding age (verum vs. sham; 59 ± 12 vs. 67 ± 13 years, *p* = 0.278), education level (13 ± 1.7 vs. 13.8 ± 3.1 years, *p* = 0.577), time of stimulation after stroke onset (49 ± 18 vs. 48 ± 27 days, *p* = 0.941), NIHSS (7 ± 8 vs. 6 ± 4, *p* = 0.675), alertness scores (257 ± 20 vs. 239 ± 32, *p* = 0.339), and performances for the picture naming task (20 ± 9.9 vs. 13.3 ± 14.2, *p* = 0.367), AAT, and ANELT (Supplementary Tables A.1–A.3). Until the end of follow-up, no significant group differences concerning language therapy frequency was observed (verum vs. sham; 23 ± 13 vs. 31 ± 11, *p* = 0.290). Regarding lesion size, the sham group tended to show larger lesions, but this difference did not reach the level of significance (46819 ± 61171 vs. 182753 ± 207984 mm^3^, *p* = 0.177) (Supplementary Table A.1 and Supplementary Figures A.4, A.5).

### Picture Naming Task

ANOVA with repeated measures revealed a significant effect of “time” [*F*(2,20) = 4.848; *p* = 0.045] but no significant “time × stimulation” interaction [*F*(2,20) = 1.038; *p* = 0.341] when the number of nouns was compared between T0, T1, and T2 and between groups. Within-group comparisons revealed significant effects of tDCS in the verum but not the sham group [*F*(2,4) = 9.478; *p* = 0.03 vs. *F*(2,4) = 1.466; *p* = 0.333]. *Post hoc* testing showed an increase of named nouns at T1 compared to T0 [*t*(5) = −4.566; *p* = 0.006] but not at T2 compared to T0 or T1 (Supplementary Table A.2). However, the number of named nouns significantly increased when the post – pre-stimulation differences between both groups were compared at T1 [*t*(10) = −3.201; *p* = 0.009] but not at T2 [*t*(10) = −0.168; *p* = 0.870] (Supplementary Figure A.3).

Regarding named verbs, no significant change was found at any time in any group (Supplementary Table A.2).

### Aachen Aphasia Test

ANOVA with repeated measures showed a significant effect of “time” [*F*(1,10) = 9.772; *p* = 0.011], but no significant interaction [*F*(1,10) = 0.879; *p* = 0.370] for the naming test of the AAT. *Post hoc* testing revealed a significant increase of the named words in the verum but not the sham group at 4 weeks after tDCS (T2) [*t*(5) = −3.058; *p* = 0.028 vs. *t*(5) = −1.464; *p* = 0.203] (Supplementary Table A.3).

Similarly, a significant effect of “time” but no “time × stimulation” interaction was observed for the number of errors of the Token Test [*F*(1,10) = 26.368; *p* = < 0.001], repetition [*F*(1,10) = 12.949; *p* = 0.005], writing [*F*(1,10) = 19.205; *p* = 0.001], comprehension [*F*(1,10) = 17.231; *p* = 0.002], and the total AAT score [*F*(1,10) = 80.121; *p* < 0.001].

Regarding the subtests of spontaneous speech of the AAT, a significant effect of “time” [*F*(1,10) = 18.000; *p* = 0.002] and a significant “time × stimulation” interaction [*F*(1,10) = 8.000; *p* = 0.018] were found for the “communication” subtest. *Post hoc* testing showed almost significant group differences at 4 weeks after tDCS (T2) [*t*(5) = −2.138; *p* = 0.058] and significant within-group effects only in the verum group [*t*(5) = −5.000; *p* = 0.004] (Supplementary Table A.3).

For the “syntactic structure” subtest, no significant effects of “time” [*F*(1,10) = 0.556; *p* = 0.473], but a significant “time × stimulation” interaction [*F*(1,10) = 5.000; *p* = 0.049] was observed with positive effects in the verum but not the sham group (Supplementary Table A.3). Conversely, for the “prosodie” subtest, a significant effect of “time” [*F*(1,10) = 22.830; *p* = 0.001], but no “time × stimulation” interaction was found.

Finally, no effect of “time” and no “time × stimulation” interaction was observed for the other subtests (“automatic speech,” “semantic structure,” and “phonematic structure”).

### Amsterdam Nijmegen Everyday Language Test (ANELT)

A significant effect of “time” [*F*(1,10) = 28.077; *p* < 0.001] but no “time × stimulation” interaction [*F*(1,10) = 0.427; *p* = 0.528] was found for the assessment of communication abilities based on the ANELT score. Within-group comparisons showed that communication improved in both the verum and sham group [*t*(5) = −3.727; *p* = 0.014 and *t*(5) = −3.858; *p* = 0.012] (Supplementary Table A.2).

### Alertness

No significant effect of time and no “time × stimulation” interaction was observed for this variable.

## Discussion

The present study investigated whether repeated daily sessions of bihemispheric tDCS over the IFG regions coupled with language therapy could improve non-fluent aphasia when performed early after stroke onset, in the postacute stage. The results were as follows: picture naming selectively improved at the end of the verum tDCS protocol, while the ANELT scores and most AAT variables improved at 4-week follow-up in both verum and sham groups, except naming and communication AAT subscores that only improved in the verum tDCS group. Finally, stimulation did not affect reaction time of the alertness task.

The present results suggest that the effects of language therapy can be enhanced by non-invasive cortical stimulation in the postacute phase of stroke by applying bihemispheric tDCS over the IFG regions with the anode on the left and the cathode on the right. So far, only three studies from the same group investigated a similar tDCS paradigm (10 or 15 sessions of bihemispheric tDCS coupled with language therapy, yet using a cross-over design), however, with chronic stroke patients (Marangolo et al., 2013b, 2014, 2016). The first of these studies assessed apraxia in eight chronic aphasic patients but showed also an improvement of language tasks lasting for 1 week (Marangolo et al., 2013b). Two recent studies (Marangolo et al., 2014, 2016) focused on chronic non-fluent aphasia and tDCS outcome related markers (resting state fMRI and BDNF levels) in seven and nine patients, respectively. These studies revealed improved speech and articulation after bihemispheric tDCS which was performed for 1 week in one of them (Marangolo et al., 2014). Hence, this promising tDCS paradigm has not been assessed in postacute stroke patients that may have a greater potential for cortical adaptive plasticity than chronic stroke patients. Our patients in the postacute stroke phase (average 50 days after stroke onset) could have further benefit from right-sided inhibitory stimulation since imaging studies of comprehension recovery showed a strong right homologous activation after at least 2 weeks and a left shifting only between 4 and 12 months after stroke in patients with good recovery (Saur et al., 2006). Studies using low-frequency (inhibitory) rTMS or cathodal tDCS of the right IFG suggest that the right shifting is rather dysfunctional (Barwood et al., 2011; Kang et al., 2011; Naeser et al., 2011; Weiduschat et al., 2011). In the present study, we aimed at supporting cortical reorganization during its natural time course by enhancing cortical excitability next to Broca’s area and by reducing dysfunctional increased cortical excitability over its homologous right area. The period of at least 1 month after stroke onset was chosen to avoid a strong interference with the spontaneous post-stroke adaptation process and also since the exact time for the left shifting may in fact depend on the site and the size of the lesion (Saur et al., 2006).

However, the assumption that bihemispheric tDCS induces stronger effects than unilateral left anodal or right cathodal tDCS has not been proven yet since no comparative study was reported so far.

Previous studies of anodal tDCS of the IFG in non-fluent aphasia showed inconsistent results which led to the absence of recommendation in international guidelines (Lefaucheur et al., 2017). Two studies of the same group showed a significant improvements of naming in chronic stroke patients with a cross-over design and 10 sessions of anodal tDCS at 1 or 2 mA for 20 min coupled with language therapy lasting for 1 month in one study (Marangolo et al., 2013a; Campana et al., 2015). Two other studies with parallel group design but postacute stroke patients (from 2 to 24 weeks; average 60 days after stroke) and 15 sessions at 1 mA and 10 min duration failed to show group differences (only within group effects) after stimulation and at 3-month follow-up (Polanowska et al., 2013a,b). It would be of interest to see whether the effects in these studies depend on the time window after stroke and whether stimulus intensity and duration would have a relevant impact on the results.

In the literature, study quality with respect to sample size, heterogeneity, and protocol design is somewhat lower for cathodal tDCS of the right IFG (Jung et al., 2011; Kang et al., 2011; Cipollari et al., 2015). These studies underline the potential benefit of right-sided inhibitory stimulation in patients with chronic aphasia and the open label study of Jung et al. (2011) showed the largest effects in the postacute phase below 30 days after stroke onset.

Combined tDCS and language therapy as used in the present study is particularly likely to improve picture naming compared to language therapy alone in addition to communication ability enhancement. This corresponds to the application of tDCS over the IFG, which is rather associated with speech production (Broca’s area, Brodman area 44 and 45) as a part of the language network (Saur et al., 2008). Only communication as part of the AAT but not as assessed in the ANELT was improved at 1 month beyond the tDCS protocol, suggesting either an influence on further parts of the network or that improved naming performance impacts on communicative abilities.

Interestingly, our results suggest that the effects of an induction phase of verum tDCS for 10 days could maintain for 1 month. This result was somewhat unexpected since previous studies in different diseases such as depression and pain suggested shorter lasting effects (see Lefaucheur et al., 2017). Thus, we assume that bihemispheric tDCS may especially enhance cortical adaptative plasticity in the postacute phase of stroke.

Some methodological issues must be addressed. First, although not significant, patients in the sham group may be slightly more severe and then less prone to rapid improvement. Larger and more homogenous groups should be expected in further studies with parallel arm design. Conversely, a crossover design appears to be not suitable in studies performed in the early phase after stroke due to the fast changing clinical picture. Second, post interventional therapeutic frequency was not controlled, which may further impact the results. Finally, non-specific cognitive effects of our tDCS protocol could be considered, especially because the IFG is relatively close to the DLPFC (F5 vs. F3), which is the usual target for tDCS studies on working memory or other cognitive variables (Kang et al., 2009; Andre et al., 2016). Actually, alertness was not modified by tDCS in our patients, but was only assessed at T2. Therefore, immediate effects on various cognitive functions cannot be completely ruled out in our study.

## Conclusion

This study shows that performing repeated sessions of bihemispheric tDCS over the IFG region during individual language therapy improves speech of non-fluent aphasic patients in the postacute stroke phase. Further studies may consider personalized stimulation protocol, taken into account lesion size and localization, as well as the respective language networks to be activated, as previously shown in an anodal tDCS study (Baker et al., 2010). Larger sample sizes and a comparison with contralateral cathodal tDCS in the early phase are warranted to support our preliminary findings.

## Author Contributions

JB, JK, RG, J-PL, SF, PE, FS, AL, and VM contributed to conception and design of the study. VM, SF, and PE organized the database. CN performed the imaging analyses. VM and SF performed the statistical analysis. VM wrote the first draft of the manuscript. SF, PE, CN, and J-PL wrote sections of the manuscript. All authors contributed to manuscript revision, read, and approved the submitted version.

## Conflict of Interest Statement

The authors declare that the research was conducted in the absence of any commercial or financial relationships that could be construed as a potential conflict of interest.
