# Supplementary figures and images for: Left Shifting of Language Related Activity Induced by Bihemispheric tDCS in Postacute Aphasia Following Stroke
Source: Front Neurosci. 2019 Apr 26;13:295. doi: 10.3389/fnins.2019.00295 (PMC6498872; doi:10.3389/fnins.2019.00295)

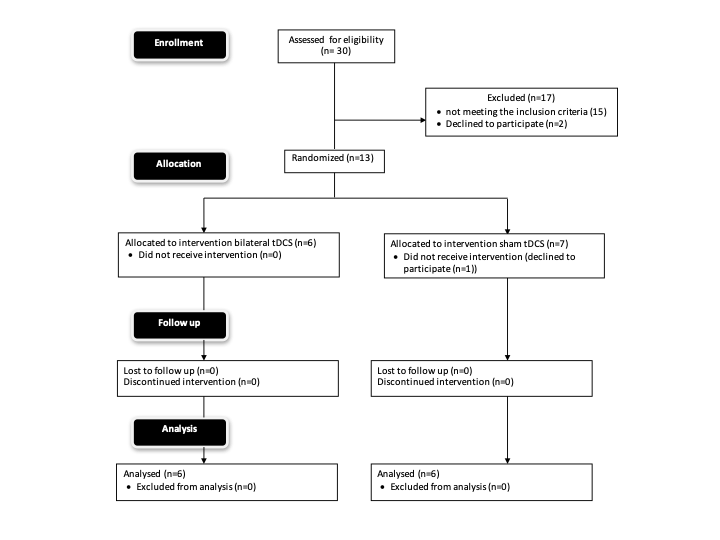

Supplement: Supplementary file 1 [file Image_1.tiff]

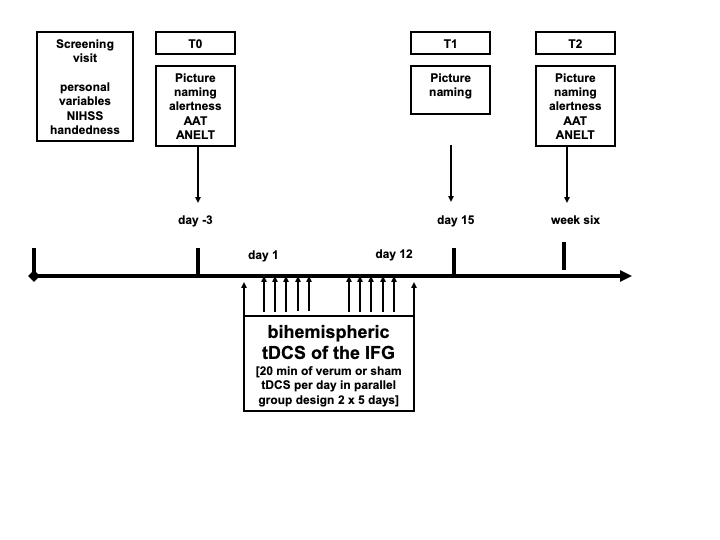

Supplement: Supplementary file 2 [file Image_2.tiff]

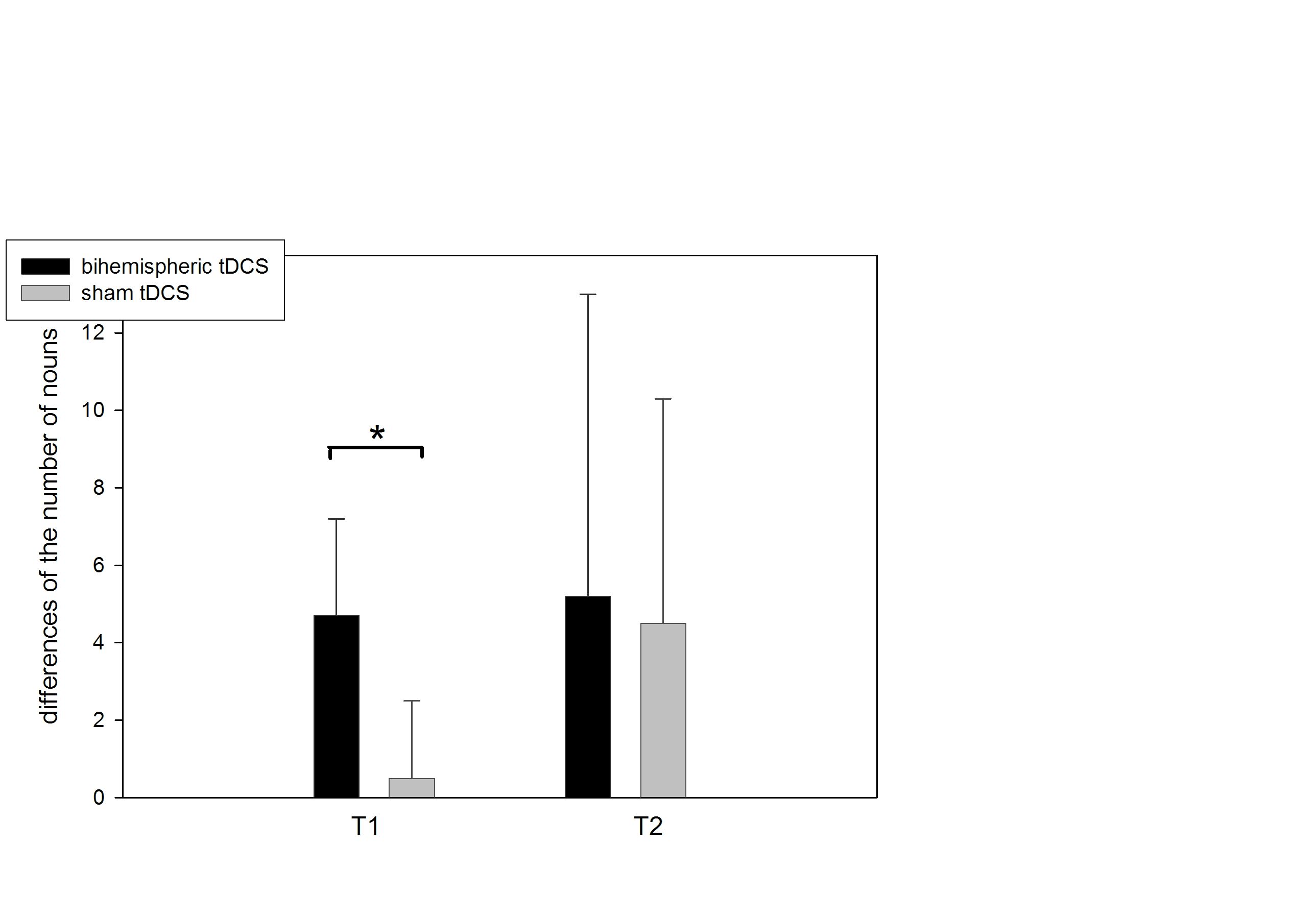

Supplement: Supplementary file 3 [file Image_3.tif]

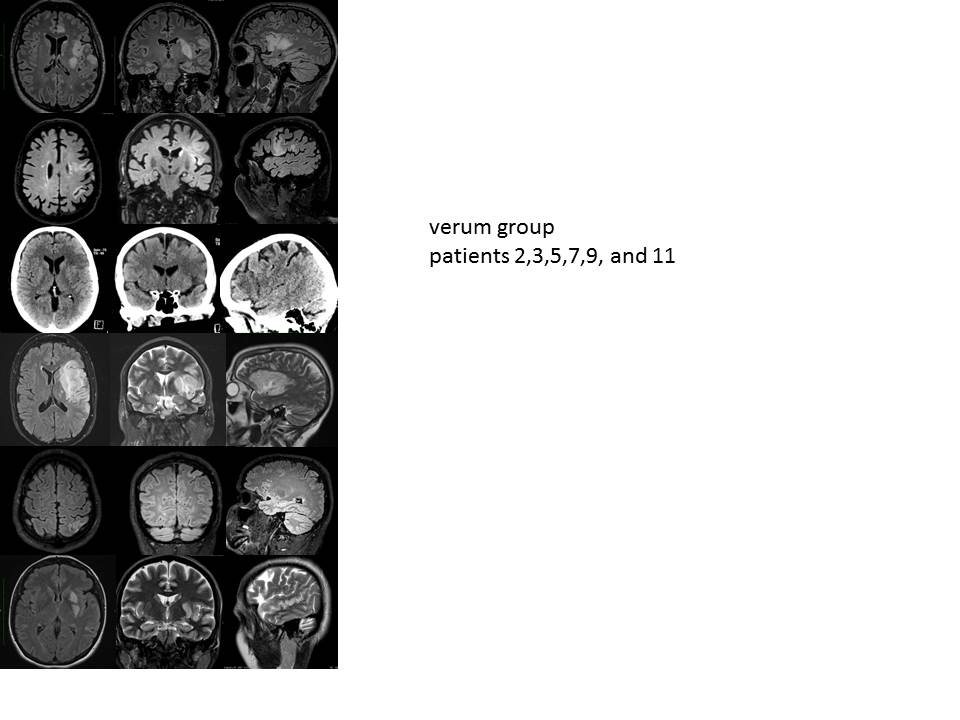

Supplement: Supplementary file 4 [file Image_4.jpg]

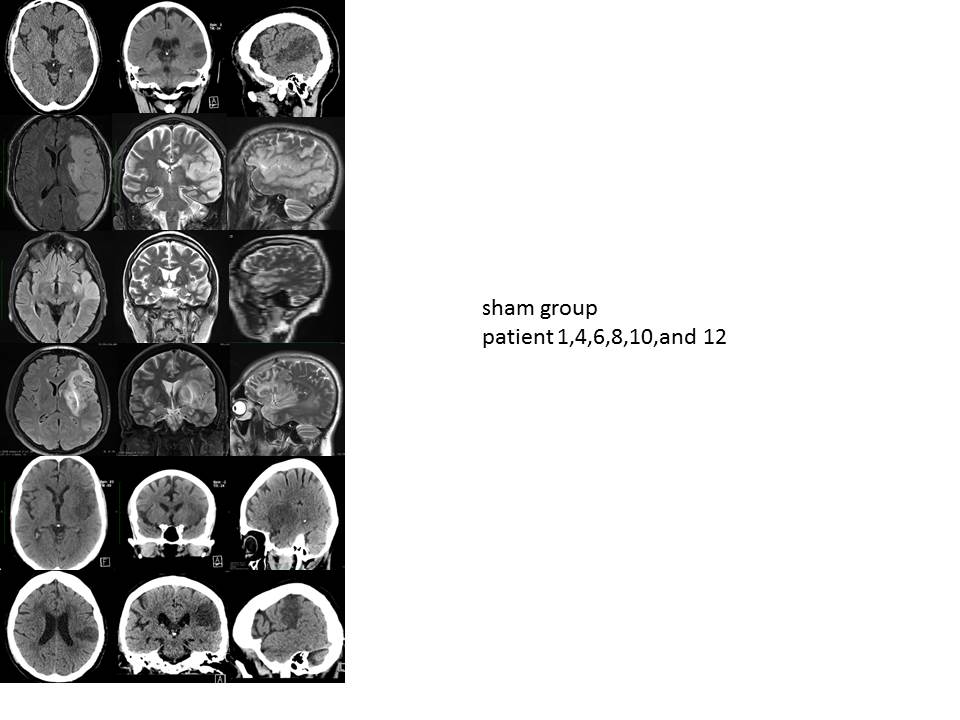

Supplement: Supplementary file 5 [file Image_5.jpg]
