# Supplementary material for: Left Shifting of Language Related Activity Induced by Bihemispheric tDCS in Postacute Aphasia Following Stroke
Source: Front Neurosci. 2019 Apr 26;13:295. doi: 10.3389/fnins.2019.00295 (PMC6498872; doi:10.3389/fnins.2019.00295)
Supplement: Supplementary file 6 [file Table_1.docx]

| Patients | Age | Sex | Education  (school+ formation) | Days after stroke at T0 | NIHSS  at T0 | Stimulation  (verum or sham tDCS) | Stroke  localization in the left hemisphere | Stroke  size in mm | Stroke  etiology | Language therapy  T1-T2 | Aphasia severity  (global AAT score) at T0 | Aphasia severity  (global AAT score) at T2 | Difference AAT  T2-T0 |
| --- | --- | --- | --- | --- | --- | --- | --- | --- | --- | --- | --- | --- | --- |
| 1 | 67 | M | 9 + 2 | 31 | 1 | S | gyrus temporalis superior, gyrus angularis, and supramarginalis (scanner) | 72x27x27 | cardial | 36 | 266 | 294 | 28 |
| 2 | 54 | M | 9 + 2 | 30 | 2 | V | multiple strokes: insular, internal capsule, and cingular gyrus | 50x30x20 | arteriosclerosis | 32 | 294 | 323 | 29 |
| 3 | 79 | M | 9 + 4 | 49 | 7 | V | Insular and frontal gyrus and postcentral gyrus | 50x20x20  17x17x20 | cardial | 4 | 237 | 252 | 15 |
| 4 | 73 | M | 9 + 4 | 95 | 9 | S | large parts of the central and peripheral media territory | 130x53x75 | arteriosclerosis | 40 | 203 | 211 | 8 |
| 5 | 61 | M | 9 + 7 | 28 | 4 | V | frontal inferior gyrus (pars triangularis and opercularis) (scanner) | 30x30x30 | dissection | 40 | 279 | 306 | 27 |
| 6 | 74 | M | 9 + 4 | 27 | 4 | S | Superior and medial temporal gyrus, and dorsal putamen | 50x40x30 | arteriosclerosis | 22 | 285 | 309 | 24 |
| 7 | 64 | F | 9 + 4 | 72 | 22 | V | frontal inferior gyrus (pars triangularis and opercularis), insular gyrus, and putamen | 84x45x45 | cardial | 16 | 276 | 291 | 15 |
| 8 | 44 | M | 9 + 4 | 36 | 11 | S | frontal inferior gyrus (pars triangularis and opercularis) and frontal medial gyrus, putamen and small part of the caudate nucleus | 57x86x76 | cardial | 40 | 199 | 220 | 21 |
| 9 | 48 | M | 9 + 3 | 46 | 4 | V | superior parietal lobe | 23x12x11 | cardial | 27 | 230 | 261 | 31 |
| 10 | 82 | M | 15 + 5 (university) | 65 | 6 | S | inferior frontal gyrus (scanner) | 50x30x30 | cardial | 32 | 233 | 240 | 7 |
| 11 | 49 | F | 9 + 4 | 66 | 3 | V | temporal superior and medial gyrus, and putamen | 40x20x20  40x15x15 | cardial | 18 | 320 | 343 | 23 |
| 12 | 64 | M | 9 + 4 | 31 | 2 | S | caudal part of the postcentral gyrus, and supramarginal gyrus (scanner) | 47x23x46 | cardial | 13 | 280 | 295 | 15 |
