# Supplementary material for: Left Shifting of Language Related Activity Induced by Bihemispheric tDCS in Postacute Aphasia Following Stroke
Source: Front Neurosci. 2019 Apr 26;13:295. doi: 10.3389/fnins.2019.00295 (PMC6498872; doi:10.3389/fnins.2019.00295)
Supplement: Supplementary file 7 [file Table_2.DOC]

**Table 2**

|  |  | **Before tDCS (T0)** | **At the end of the tDCS protocol (T1)** | **T0 vs. T1 (p value)** | **At 4 weeks after tDCS (T2)** | **T0 vs. T2 (p value)** |
| --- | --- | --- | --- | --- | --- | --- |

| **Bihemispheric tDCS** | PNT-nouns | 20.0 ± 9.9 | 24.7 ± 8.9 | **0.006** | 25.2 ± 4.7 | 0.165 |
| --- | --- | --- | --- | --- | --- | --- |
|  | PNT-verbs  ANELT | 15.3 ± 8.3  21.8 ± 13.2 | 18.2 ± 6.8 | 0.179 | 21.3 ± 5.0  28.7 ± 10.6 | 0.116  **0.014** |
| **Sham tDCS** | PNT-nouns | 13.3 ± 14.2 | 13.8 ± 13.0 | 0.562 | 17.8 ± 11.7 | 0.117 |
|  | PNT-verbs  ANELT | 11.5 ± 12.7  13.5 ± 12.7 | 10.8 ± 11.7 | 0.394 | 12.0 ± 11.9  18.8 ± 15.0 | 0.737  **0.012** |

**Significant results are marked as bold**
