# Supplementary material for: Left Shifting of Language Related Activity Induced by Bihemispheric tDCS in Postacute Aphasia Following Stroke
Source: Front Neurosci. 2019 Apr 26;13:295. doi: 10.3389/fnins.2019.00295 (PMC6498872; doi:10.3389/fnins.2019.00295)
Supplement: Supplementary file 8 [file Table_3.DOC]

**Table 3**

|  |  | **Before tDCS (T0)** | **At 4 weeks after tDCS (T2)** | **T0 vs. T2 (p value)** |
| --- | --- | --- | --- | --- |

| **Bihemispheric tDCS** | Token test  AAT repetition  AAT writing  **AAT naming**  AAT comprehension  **AAT communication**  **AAT** prosodie  **AAT** automatic speech  **AAT** semantic  **AAT** phonematic  **AAT** syntactic  **AAT overall** | 55.5 ± 6.9  53.8 ± 9.2  56.0 ± 9.1  52.7 ±10.7  54.7 ± 5.7  2.3 ± 1.2  3.8 ± 1.8  3.8 ± 1.5  3.0 ± 1.1  3.8 ± 1.5  2.5 ± 1.1  272.7 ±34.2 | 60.3 ± 7.8  57.3 ± 6.0  58.3 ± 8.3  59.2 ±10.9  60.8 ± 8.0  3.2 ± 1.0  4.2 ± 1.3  4.5 ± 0.8  3.3 ± 0.5  4.0 ± 1.3  3.5 ± 1.4  296.0 ±35.3 | **0.005**  0.076  **0.022**  **0.028**  **0.038**  **0.004**  0.175  0.235  0.465  0.611  **0.041**  **<0.001** |
| --- | --- | --- | --- | --- |

| **sham**  **tDCS** | Token test  AAT repetition  AAT writing  **AAT naming**  AAT comprehension  **AAT communication**  **AAT** prosodie  **AAT** automatic speech  **AAT** semantic  **AAT** phonematic  **AAT** syntactic  **AAT overall** | 51.0 ±14.2  46.5 ± 4.9  49.7 ± 8.6  45.0 ± 7.9  52.2 ± 7.8  1.7 ± 0.8  3.5 ± 1.9  3.5 ± 2.0  2.2 ± 1.5  1.5 ± 0.8  2.8 ± 1.7  244.3 ± 38.2 | 57.2 ±15.6  48.8 ± 5.3  51.7 ± 8.8  48.5 ±10.6  55.3 ± 7.3  1.8 ± 1.2  3.5 ± 1.9  3.5 ± 2.0  2.5 ± 1.9  2.2 ± 1.7  2.3 ± 1.6  261.5 ± 42.8 | **0.022**  **0.003**  **0.033**  0.203  **0.001**  0.363  1.00  1.00  0.363  0.235  0.415  **0.005** |
| --- | --- | --- | --- | --- |

**Significant results are marked as bold**
