# Supplementary material for: Left Shifting of Language Related Activity Induced by Bihemispheric tDCS in Postacute Aphasia Following Stroke
Source: Front Neurosci. 2019 Apr 26;13:295. doi: 10.3389/fnins.2019.00295 (PMC6498872; doi:10.3389/fnins.2019.00295)
Supplement: Supplementary file 9 [file Data_Sheet_1.docx]

**Appendix A**

**Fig. A.1 Flow-chart of the study**

**Fig. A.2 Experimental protocol design**

NIHSS: National Institute of health stroke scale; AAT: Aachen Aphasia test; ANELT: Amsterdam Nijmegen Everyday Language Test; tDCS: transcranial direct current stimulation; IFG: inferior frontal gyrus.

**Fig. A.3 Differences of the number of nouns of the picture naming task compared to baseline**

**Fig. A.4. and A.5. Neuroimaging in the acute stroke stage**

MRI or scanner imaging of all patients in the axial, coronal, and saggital plane used for lesion size calculation. A.4. verum group A.5. sham group

**Table A.1 Patients characteristics**

NIHSS: National Institute of health stroke scale; AAT: Aachen Aphasia test; tDCS: transcranial direct current stimulation

**Table A.2 Results of the picture naming task and the ANELT**

PNT: picture naming task; ANELT: Amsterdam Nijmegen Everyday Language Test; tDCS: transcranial direct current stimulation

**Table A. 3 Results of the AAT**

AAT: Aachen Aphasia test; tDCS: transcranial direct current stimulation
